# Sierra Nevada sweep: metagenomic measurements of bioaerosols vertically distributed across the troposphere

**DOI:** 10.1038/s41598-020-69188-4

**Published:** 2020-07-24

**Authors:** Crystal Jaing, James Thissen, Michael Morrison, Michael B. Dillon, Samantha M. Waters, Garrett T. Graham, Nicholas A. Be, Patrick Nicoll, Sonali Verma, Tristan Caro, David J. Smith

**Affiliations:** 1 Physical & Life Sciences Directorate, Lawrence Livermore National Laboratory, Livermore, CA USA; 2 Universities Space Research Association, Maryland, USA; 3 Georgetown University Medical Center, Washington, DC USA; 4 University of Victoria, Victoria, BC Canada; 5 Blue Marble Space Institute of Science, Space Bioscences Division, NASA Ames Research Center, Moffett Field, CA USA; 6 Department of Geological Sciences, University of Colorado, Boulder, CO USA; 7 NASA Ames Research Center, Space Biosciences Division, Moffett Field, CA USA

**Keywords:** Biological techniques, Microbiology, Environmental sciences

## Abstract

To explore how airborne microbial patterns change with height above the Earth’s surface, we flew NASA’s C-20A aircraft on two consecutive days in June 2018 along identical flight paths over the US Sierra Nevada mountain range at four different altitudes ranging from 10,000 ft to 40,000 ft. Bioaerosols were analyzed by metagenomic DNA sequencing and traditional culturing methods to characterize the composition and diversity of atmospheric samples compared to experimental controls. The relative abundance of taxa changed significantly at each altitude sampled, and the diversity profile shifted across the two sampling days, revealing a regional atmospheric microbiome that is dynamically changing. The most proportionally abundant microbial genera were *Mycobacterium* and *Achromobacter* at 10,000 ft; *Stenotrophomonas* and *Achromobacter* at 20,000 ft; *Delftia* and *Pseudoperonospora* at 30,000 ft; and *Alcaligenes* and *Penicillium* at 40,000 ft. Culture-based detections also identified viable *Bacillus zhangzhouensis*, *Bacillus pumilus*, and *Bacillus* spp. in the upper troposphere. To estimate bioaerosol dispersal, we developed a human exposure likelihood model (7-day forecast) using general aerosol characteristics and measured meteorological conditions. By coupling metagenomics to a predictive atmospheric model, we aim to set the stage for field campaigns that monitor global bioaerosol emissions and impacts.

## Introduction

Aerosols (mostly desert dust, black carbon and ocean spray) regularly disperse across the Pacific Ocean with springtime atmospheric winds – in fact, models suggest that as much as 64 Teragrams of Asian aerosols can be transported to North America annually^1^. Past studies have reported microorganisms co-transported with globally-dispersed aerosols, and that both DNA signatures and viable cells can be detected in air masses^2–4^. For example, thousands of taxonomic signatures were measured in springtime Asian dust plumes delivering free tropospheric bioaerosols to an alpine research station in central Oregon^3^. In a separate flight project surveying airborne microorganisms in the western region of the United States, several taxa were found to be enriched in the upper atmosphere, including bacterial families *Lachnospiraceae*, *Ruminococcaceae* and *Erysipelotrichaceae*^2^. Despite harsh conditions in the atmosphere, viable bacterial isolates were recovered from the same study^2^, including *Bacillus* sp., *Micrococcus* sp., *Arthrobacter* sp. and *Staphylococcus* sp.

Previous aerobiology campaigns using aircraft were flown at single altitudes in order to refine collection methods and demonstrate some of the first measurements of an atmospheric microbiome^2,5–7^. To date, there are only a few studies that have examined microbiological changes across a vertical gradient in Earth’s troposphere^8–10^. More studies using advanced genomic techniques such as shotgun metagenomic sequencing will be essential to formulating predictive models of bioaerosols and how the atmospheric microbiome is influenced by meteorological conditions^11,12^. High-altitude aircraft can be used to travel at variable altitudes in the atmosphere, allowing us to investigate how bioaerosols patterns change with height above the surface. Herein, we report microbial composition in a “staircase pattern” flown at 10,000 ft, 20,000 ft, 30,000 ft and 40,000 ft using an Aircraft Bioaerosol Collector (ABC) device onboard NASA’s C-20A aircraft^2^. Air samples were collected on two consecutive flight days (June 20–21, 2018) in the vicinity of Palmdale, CA, USA, across the Sierra Nevada mountain range. Following each flight, captured bioaerosols were analyzed with traditional culture-based methods as well as metagenomic DNA sequencing. A meteorological and atmospheric dispersion computer model was then applied to the microbiological dataset to provide predictions of bioaerosol measurement signals and potential population exposures in downwind regions.

## Methods

### Sampling flights

**Figure 1:**
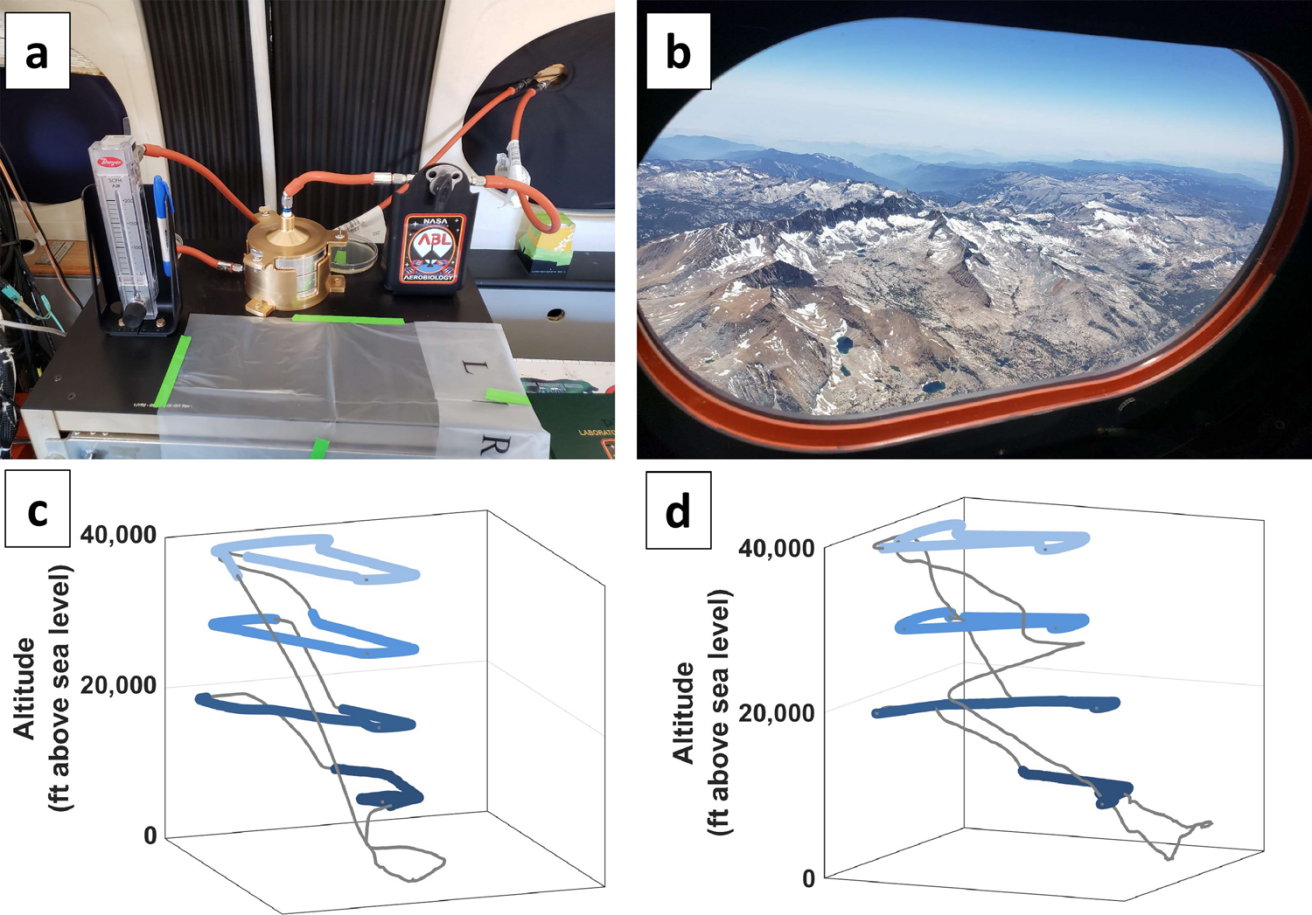
Sampling over the US Sierra Nevada mountain range. (**a**) Aircraft Bioaerosol Collector (ABC) onboard NASA’s C-20A aircraft. Air sampling line, flow valve, cascade impactor, and air flow meter shown on workbench; (**b**) June 20, 2018, view from C-20A window at lowest altitude sampled (10,000 ft) above the US Sierra Nevada mountain range with an aerosol haze visible on the horizon; (**c**) Flight pattern on June 20, 2018 based on GPS data, sampling for 30–40 min at 10,000 ft steps up to 40,000 ft; (**d**) Flight pattern on June 21, 2018 based on GPS data, sampling for 30–40 min starting at 40,000 ft and stepping down in 10,000 ft steps. Panels C and D were generated using Mathworks Matlab software, versions R2018 to R2019 (https://www.mathworks.com/products/matlab.html), from the recorded flight path.

Two research flights (Fig. 1) were flown over the Sierra Nevada mountains (Supplementary Fig. S1) across California using NASA’s C-20A Gulfstream III aircraft available through the Airborne Science Program at Armstrong Flight Research Center (AFRC). The first flight was 2.9 h total flown on June 20, 2018; the second flight was 3.5 h total on June 21, 2018. During each daytime flight, the aircraft ascended to 40,000 ft and remained at this altitude for 30–40 min sampling air at an estimated rate of 8.5 L·min^−1^. Then, in a “staircase” pattern, the aircraft descended in 10,000 ft increments from 30,000 ft, to 20,000 ft, to 10,000 ft, for 30–40 min of air sampling at each altitude step. Supplementary Table S1 summarizes sampling times and other pertinent flight information. Aircraft telemetry data was visualized using Google Earth Pro software with images from Landsat and Copernicus to generate terrain (Supplementary Fig. S1).

Sample collection procedures were carried out using the ABC hardware system as previously described^2^. Before the aircraft reached each sampling altitude, gamma-irradiated (i.e., DNA-treated) gelatinous filter membranes were loaded into a cascade impactor (Supplementary Fig. S2). Two different cascade impactor stages were used to size separate the bioaerosols (Product TE-10–860; Tisch Environmental, Cleves, OH). Each sampler stage had 400 small, round, drilled orifices (1.18 mm on first stage; 0.25 mm on second stage). As described by Smith et al.^2^, the first stage should capture aerosols larger than 8 μm (if traveling into the inlet) while the second stage would capture smaller aerosols between 1 and 8 μm (nominal). The first stage holds Filter A and second stage holds Filter B. An upstream valve was used to regulate atmospheric air flowing into the system. With the air flow off, fresh filters were aseptically swapped into the sampling system before the next altitude was sampled. A total of 16 filter samples were collected at the four different altitudes on two consecutive flight days with two filters staged at each altitude.

### Experimental controls

To produce a baseline for assessing known system contaminants, we followed a previously described method^2^where pre-flight and post-flight *ground controls* were collected on the C-20A window plate, air probe inlet, and an exterior portion of the aircraft upstream of the sampling probe using a pre-wetted, DNA-free sterile applicator (Puritan cat #25-3306UTTFDNA). Swabs were stored in 5 mL of sterile deionized water within a 15 ml Falcon tube and kept at 4 °C until laboratory processing. To characterize contaminants inside the aircraft, a blank gelatinous filter was placed in a petri dish resting on the work bench and exposed to ambient aircraft cabin air during every filter exchange. These samples were reported as *cabin controls*. The surface of the bench holding the cascade sampler was also swabbed with a pre-wetted, DNA-free sterile applicator as an additional *cabin control*.

### Post-flight sample processing

For each sample, one filter half was archived in a −80 °C freezer (TSU-600A, Thermo Scientific, Asheville, NC) and the other half was put in 40 ml of warm (37 °C) molecular grade water which easily dissolved the gelatinous filter membrane. The sample was then concentrated using the InnovaPrep CP Select concentrating pipette (InnovaPrep, Drexel, MO). The concentrator passed the entire sample volume (40 ml) through a 0.1 µm flat polyethersulfone membrane (part number CC08001), followed elution buffer (EB, phosphate buffering solution with 0.75% Tween-20 [PBST]) into a final output volume of 1 mL. Filters A and B were processed separately. Similarly, swabs contained in 15 mL tubes (wetted with 5 ml of sterile water) from ground and cabin control samples were concentrated in 1 mL of EB. For each concentrated volume, 200 µL was used for DNA extraction and subsequent metagenomic sequencing; 100 µL was used for culture-based recovery assays; and 700 µL was archived at −80 °C in the freezer in 20% glycerol stocks (final volume). We have included three *process controls*. The first one is the whole blank gelatinous membrane filter that has gone through the entire InnovaPrep processing and DNA extraction; the other two are two replicates of the DNA extraction with no template controls. A total of 13 ground, cabin and process control samples were collected during the aircraft flight operations. Supplementary Table S2 summarizes all experimental samples.

### DNA extraction

A total volume of 200 µl was utilized for DNA extraction from the 1 mL InnovaPrep concentrated sample. Each sample was extracted using the Qiagen AllPrep PowerViral DNA/RNA Kit (part number 28000, Hilden, Germany). Standard manufacturer’s protocols with bead beating were followed to extract DNA from each sample. Samples were eluted in 60 µL of elution buffer and quantitated using the ThermoFisher Qubit Fluorometer (Waltham, MA). The average concentration of flight samples and controls was approximately 14.9 pg/µL.

### Metagenomic sequencing

The Illumina NextSeq500 was used for shotgun metagenomic sequencing with the Illumina NextSeq Series High Output Kit v2 (San Diego, CA), using 150 base pair, paired-end reads^13^. DNA libraries were prepared for sequencing using the Illumina Nextera Flex DNA Library Preparation Kit (part number 20018705). For each sample, approximately 400 pg was input into the kit and standard manufacturer protocols were followed. Quality, concentration, and fragment sizes of the completed libraries were assessed on the Agilent Tapestation 4200 (Santa Clara, CA). Libraries were normalized and pooled to 100–200 pM according to the Agilent Tapestation determined concentrations. The pooled library sample was quantitated using the ThermoFisher Qubit fluorimeter and denaturing steps were performed according to the manufacturer’s standard recommendations (Illumina). A concentration of 1% of Illumina’s standard positive control (PhiX) was added to the pooled libraries and 1.3 pM final library concentration was input into the Illumina NextSeq 500 instrument.

### Taxonomy identification

Metagenomic sequences were analyzed using the Livermore Metagenomics Analysis Toolkit (LMAT), a metagenomic analysis pipeline that searches for taxonomic identifiers associated with k-mers in reference genome databases^14,15^. The database contains distinct species-level alignments for 4,863 bacteria; 4,189 viruses; 2,038 eukaryotes; and 279 archaea.

### Ecological distance

Similarity between samples was visualized using non-metric multidimensional scaling (NMDS) and the t-Distributed Stochastic Neighbor Embedding (tSNE) methods. NMDS analysis was performed by Jaccard distance of detection, comparing microbial presence (above 1 LMAT read) and absence differences at the genus level between samples. tSNE of quantification matrices was used to reduce high-dimensional data into a low-dimensional space for visualization^16^. Through both data processing techniques, samples with high similarity will cluster together whereas less similar samples will be spaced further apart. Prior to tSNE analyses, the relative abundance of each taxon in a sample was transformed into Euclidian space using the center log-ratio (clr) method in the ALDEx2 (v.1.16.0) R package^17^.

### Alpha diversity and differential abundance

Alpha diversity refers to the diversity within an ecosystem. It is usually expressed by the number of species (i.e., species richness) in that ecosystem. Alpha (α) diversity was estimated for each sample and compared within altitudes across flight days. We quantified α-diversity using the Chao1 estimator^18^ and the observed number of effective taxa (^q^N_eff_) weighted by each taxonomic proportion of DNA per sample^19^. Weights were chosen such that ^q^N_eff_ corresponded to the following transformations of commonly used diversity indices: observed richness (the number of taxa seen; ^0^N_eff_), exponentiated Shannon index (both taxa richness and evenness; ^1^N_eff_), and the reciprocal Simpson index (dominance and evenness of taxa; ^2^N_eff_).

A differential abundance analysis was performed for taxa at each altitude step using the ALDEx2 (v.1.16.0) R package^17^. Individual abundances for an identified taxon were transformed using the centered log-ratio (clr) method^17,20^. Transformed counts were then analyzed for differential abundances across altitudes steps using the aldex.glm() function. Finally, *P* values for taxa were corrected for multiple tests using the Benjamini and Hochberg method^21^.

### Culture-based recovery and identification

After sample concentration, 100 µL aliquots were spread onto Reasoner's 2A agar (R2A) plates and Trypticase soy agar (TSA) plates, wrapped in Parafilm (American National Can, Chicago, IL) and incubated in the dark at 25 °C for up to four weeks while monitoring signs of growth. Individual colonies were sub-cultured on R2A until isolated, and then cryopreserved with 10% sterile glycerol (Amresco, Solon, OH) and nutrient broth (Difco, Sparks, MD) at -80 °C. Bacterial colonies DNA extraction and 16S rRNA Sanger sequencing was done by GENEWIZ (South Planfield, NJ). Forward and reverse 16S rRNA sequences were merged and searched against the NCBI non-redundant database using BLASTn^22^.

### Atmospheric modeling

HYSPLIT back-trajectories^23^were obtained using reanalysis data on the READY website, https://www.ready.noaa.gov. Heights at the different sampling altitudes were used as input for starting points in the model.

Atmospheric microbes are co-transported in aerosols that have a wide range of sizes^24,25^. We forecasted aerosol (forward) dispersal from the sampled locations using several modeling process assumptions. Since the typical lifetime of relatively small particles in the upper troposphere (e.g., 10 days for 9 μm particles^26^) is comparable to the analysis timescale of one week, our analysis does not require a detailed particle size distribution. First, the bioaerosols were assumed to be 1-μm aerodynamic diameter particles released at a constant rate along the sampling flight path. Our choice of a spatially and temporally uniform particle concentration matches the sampling resolution within the flight path. Second, the downwind time-integrated concentration of the released material was predicted using the Department of Energy, Lawrence Livermore National Laboratory, National Atmospheric Release Advisory Center (DOE LLNL NARAC) modeling system. Atmospheric Data Assimilation and Parameterization Techniques (ADAPT) / Lagrangian Operational Dispersion Integrator (LODI) models. The ADAPT model was initialized with 0.25 deg resolution Global Forecast Model fields^27,28^. Our modeling resolved North American near-surface influences up to seven days after flights with a 30.8 km horizontal resolution. Default ADAPT upper-tropospheric dispersion parameters were used: 0.01 m^2^·s^−1^ and 0.25 m^2^·s^−2^ for the vertical eddy diffusivity (K_z_) and horizontal velocity variance (σ_u_^2^ = σ_v_^2^), respectively, and, while reasonable for many situations, may underestimate the overall vertical atmospheric mixing during the seven day period. Non-precipitation atmospheric removal processes were accounted for by including both (a) gravitational settling and (b) an additional 0.003 m·s^−1^ deposition velocity. We chose the latter value to reflect deposition to regions with significant vegetation^29^. In order to simplify the modeling effort, other known effects on aerosol lifetimes in the atmosphere, including highly convective conditions and additional emissions from the Earth’s surface were not considered. Finally, to make our forecasts more relevant to downwind human population centers, we estimated the “average” outdoor individual and population-level exposures. The individual exposure probability was calculated by multiplying (a) predicted near-surface outdoor aerosol concentrations by (b) an average adult breathing rate (respiratory second volume) of 2 × 10^–4^ m^3^·s^−1^^30^. The population-level exposure estimate was calculated by multiplying the individual exposure estimate by the population as reported in the LandScan 2015 High Resolution Global Population Database^31^.

## Results

### Metagenomics

**Figure 2:**
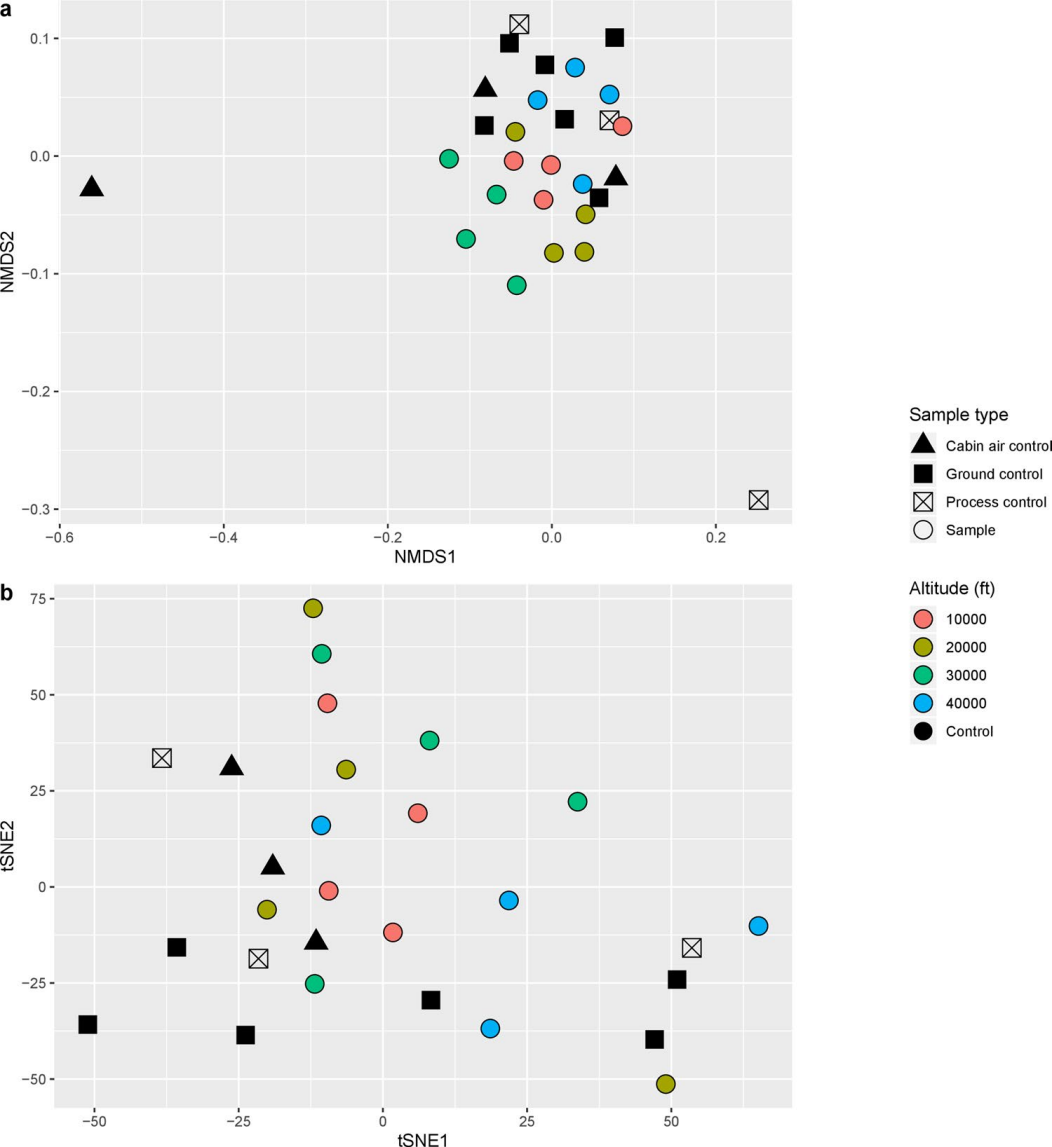
Visualization of sample clustering using multiple distance and similarity metrics. (**a**) Non-metric multidimensional scaling (NMDS) using the Jaccard distance depicting sample similarity with taxonomic presence-absence profiles; (**b**) Sample clustering using t-Distributed Stochastic Neighbor Embedding (tSNE) analysis of taxa abundance. The figure was generated in R (v.3.6.0) ^53^ using package ggplot2 (v.3.2.1)^54^.

Approximately 10 M reads were obtained for each flight sample. After LMAT analysis, which combines forward and reverse reads into a single unit, an average of 5 M reads per sample were uniquely assigned to a taxonomic identifier at the given score threshold^14,15^. On average, 940,000 reads per sample corresponded to microbial sequences, out of which 613,000 reads were assigned at the genus level. The detailed number of reads for each flight and control sample is listed in Supplementary Table S2. Two statistical methods were used to visualize sample clustering: NMDS and tSNE. Neither of the two data visualization approaches showed clear clustering of flight samples by altitude (Fig. 2). Colored circles represent flight samples from each altitude. Four samples from each altitude were included: filters A and B from Day 1 and filters A and B from Day 2. Three cabin air control, three process control and six ground control samples were included in both data visualization methods to help determine if atmospheric samples were significantly different from the controls. Jaccard distance (Fig. 2a) showed a tighter grouping of the flight samples based on altitude, but overlap patterns suggested a high amount of similarity between sampling at different heights. Most of the control samples clustered together by NMDS visualization (Fig. 2a); tSNE also showed majority of controls separated from flight samples (Fig. 2b).

### Alpha diversity

**Figure 3:**
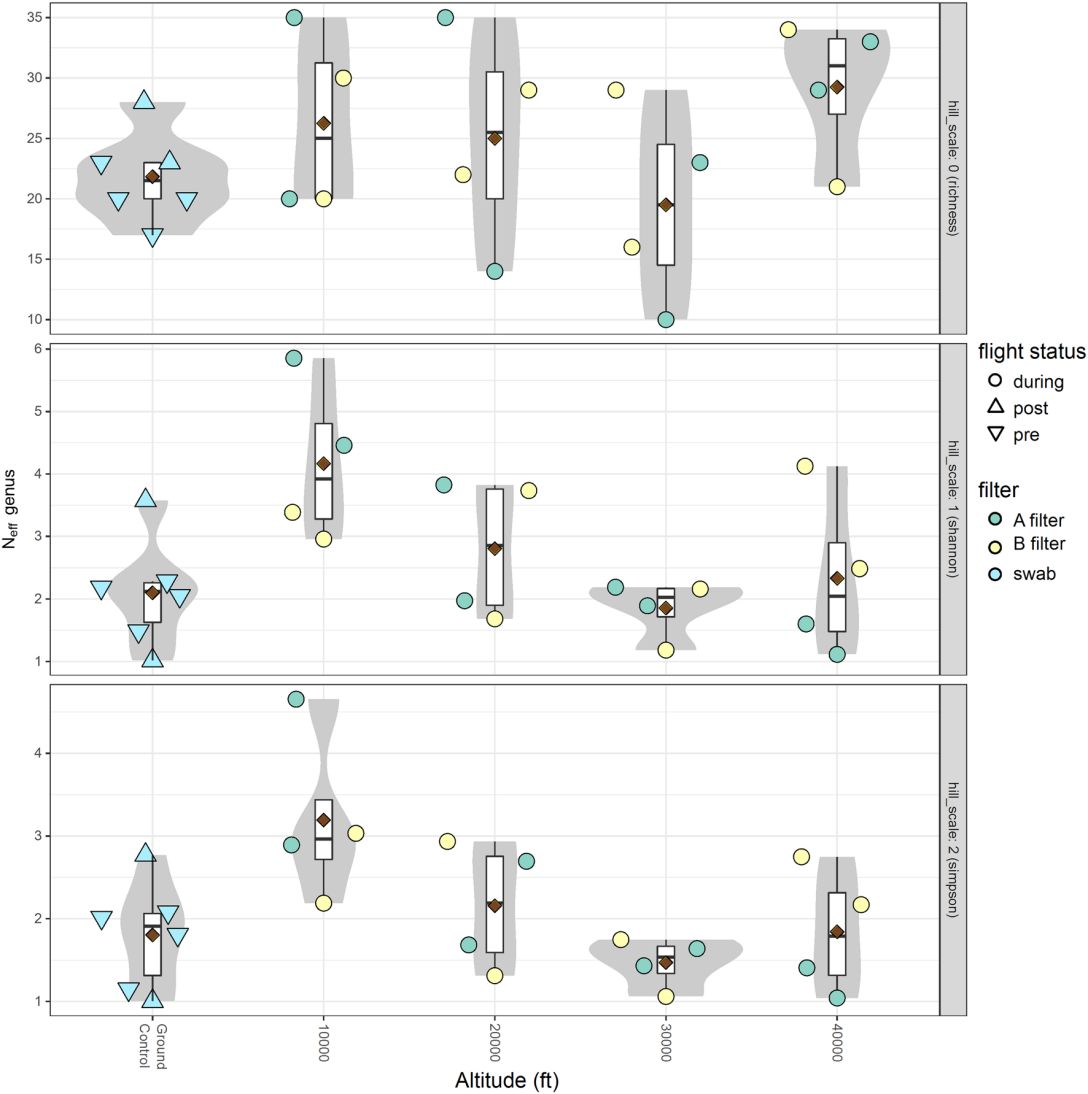
Alpha (α)-diversity for flight and ground samples at genus level resolution. α-diversity quantified as ^1^N_eff_ with samples shown as circles. The area of each circle is proportional to the number of genus-resolved fragments in a sample. Violin plots show density of α-diversity, box plots show the first, second and third quartiles, and 1.5 times the interquartile range of each time point’s α-diversity sample distribution. Dark red diamonds represent the means. The figure was generated in R (v.3.6.0)^53^ using package ggplot2 (v.3.2.1)^54^.

Figure 3 is a genus level comparison of richness and evenness between samples (ground and flight); Supplementary Fig. S3 shows the alpha diversity at the species level. Six ground controls (pre-flight and post-flight swabs), along with four data points from each altitude (Filters A (alpha) and B (beta); both flight days) were included in the diversity comparison. No significant differences between samples were observed using a Kruskall-Wallis one-way ANOVA of species richness, nor Shannon and Simpson indices. Further pairwise comparisons of ground, 10,000 ft and 30,000 ft groups using the Mann–Whitney and Student’s t-tests found no significant difference between the groups either. The Simpson index comparison at the genus level resulted in the smallest *p* value of 0.078.

### Relative abundance

**Figure 4:**
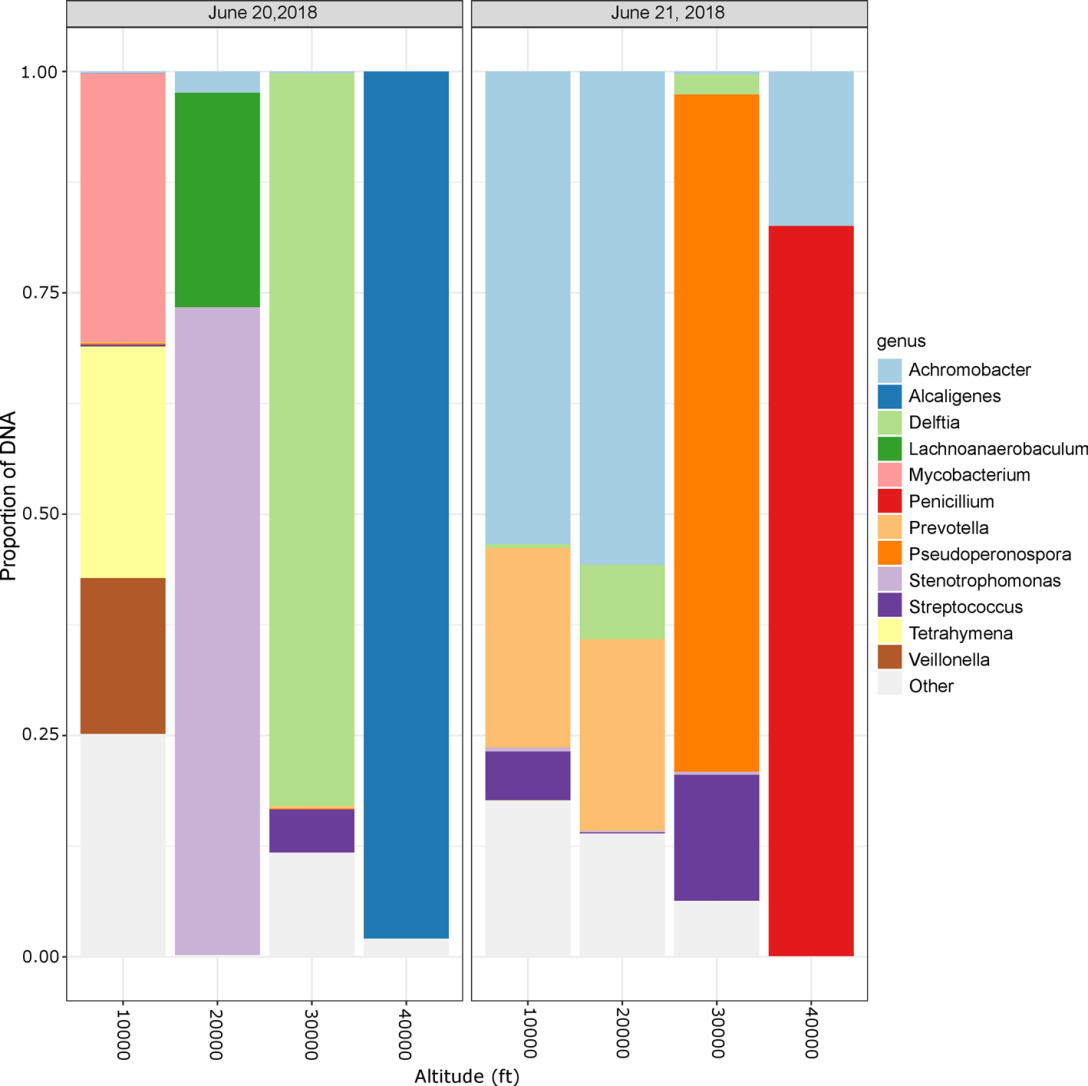
Relative abundance of genera across altitudes on both sampling days. Samples from Filter A separated by day and collection altitude to examine shifts in relative abundance of genera. *Achromobacter, Stenotrophomonas, Streptococcus,* and *Delftia* were among the top 12 most abundant taxa in both days*.* Bacterial genera that were not among the top 12 represented by light gray colored bars. In additional to bacterial and fungal taxa shown, *Pinus koraiensis* and *Pinus radiata* were also detected at 10,000 ft on the second flight day (June 21, 2018) but were not plotted for clarity. The figure was generated in R (v.3.6.0)^53^ using package ggplot2 (v.3.2.1)^54^.

Proportionally abundant microbial taxa from each flight day are depicted for Filter A (Fig. 4) and Filter B (Supplementary Fig. S4). The 12 most abundant genera in Filter A (from either day) were *Achromobacter, Alcaligenes, Delftia, Lachnoanaerobaculum, Mycobacterium, Penicillium, Prevotella, Pseudoperonospora, Stenotrophomonas, Streptococcus, Tetrahymena,* and *Veillonella.*. Detected as proportionally abundant in both days were *Achromobacter, Stenotrophomonas, Streptococcus,* and *Delftia.* On June 20, 2018, *Lachnoanaerobaculum*, *Alcaligenes, Mycobacterium*, *Tetrahymena,* and *Veillonella* appeared, whereas *Penicillium, Prevotella, Pseudoperonospora,* and *Porphyromonas* were only abundant on June 21, 2018. In addition to microbial taxa, *Pinus koraiensis* and *Pinus radiata* were also detected as proportionally abundant at 10,000 ft but only on the second flight day (June 21, 2018). Species level abundance of Filter A is depicted in Supplementary Fig. S5, *Achromobacter xylosoxidans*, *Alcaligenes faecalis*, *Bradyrhizobium* sp. DFCI-1, *Delftia acidovorans*, *Delftia* sp. Cs1-4, *Lachnoanaerobaculum saburreum*, *Mycobacterium abscessus*, *Paenibacillus fonticola*, *Penicillium aurantiogriseum*, *Pseudoperonospora cubensis*, *Stenotrophomonas maltophilia*, and *Veillonella dispar* were detected. The top 12 most abundant genera in Filter B included *Achromobacter, Bradyrhizobium, Byssochlamys, Chryseobacterium, Delftia, Enterobacter, Gardnerella, Meiothermus, Penicillium, Prevotella, Stenotrophomonas, Streptococcus* (Supplementary Fig. S3).

In the experimental controls (Supplementary Fig. S6), the most abundant detected species or plasmids were *Achromobacter xylosoxidans*, *Acidovorox sp.* JS42 plasmid pAOVO01, *Alicycliphilus denitrificans*, *Aspergillus oryzae*, *Aureobasidium pullulans*, *Bradyrhizobium* sp. DFCI-1, *Capnocytophaga sp.* oral taxon 326*, Eimeria mitis*, *Eimeria praecox*, *Haemophilus parainfluenzae*, *Neosartorya fischeri*, and *Prevotella sp.* ICM33. *Achromobacter xylosoxidans* and *Bradyrhizobium* sp. DFCI-1 were detected in both flight samples and control samples, suggesting the possibility of contamination associated with those reads.

Table 1 shows the taxonomic distinctions between samples, summarizing the five most abundant genera from each altitude and controls (depicting only Filter A samples for clarity). At 10,000 ft, the distribution of the top five genera was even (with abundances ranging from 30 to 9% on June 20, 2018; and 53% to 4% on June 21, 2018). At 40,000 ft, the top five taxa were dominated by a single genus (e.g., *Alcaligenes* 97% on June 20, 2018; *Penicillium* 82% on June 21, 2018). The altitude where the taxonomic composition was most similar between the two days sampled was 30,000 ft where both *Delftia* and *Streptococcus* were proportionally abundant.

**Table 1:** Top five most abundant genera at each altitude compared to pre-flight ground controls.

Altitude (ft)	June 20, 2018		June 21, 2018	
	Genus	Relative abundance (%)	Genus	Relative abundance (%)
0	*Eimeria*	93.29	*Prevotella*	26.46
0	*Delftia*	1.81	*Alicycliphilus*	22.63
0	*Penicillium*	0.99	*Achromobacter*	19.53
0	*Solirubrobacter*	0.88	*Streptococcus*	19.20
0	*Escherichia*	0.77	*Acidovorax*	10.11
10,000	*Mycobacterium*	30.37	*Achromobacter*	53.37
10,000	*Tetrahymena*	26.16	*Prevotella*	22.52
10,000	*Veillonella*	17.60	*Escherichia*	6.46
10,000	*Bradyrhizobium*	12.22	*Streptococcus*	5.51
10,000	*Orpinomyces*	8.98	*Porphyromonas*	4.14
20,000	*Stenotrophomonas*	73.11	*Achromobacter*	55.71
20,000	*Lachnoanaerobaculum*	24.22	*Prevotella*	21.63
20,000	*Achromobacter*	2.39	*Delftia*	8.40
20,000	*Chryseobacterium*	0.22	*Aspergillus*	5.80
20,000	*Astrammina*	0.01	*Helicobacter*	5.74
30,000	*Delftia*	82.72	*Pseudoperonospora*	76.50
30,000	*Paenibacillus*	10.87	*Streptococcus*	14.24
30,000	*Streptococcus*	4.88	*Pseudomonas*	6.31
30,000	*Chryseobacterium*	0.30	*Delftia*	2.25
30,000	*Prevotella*	0.30	*Stenotrophomonas*	0.34
40,000	*Alcaligenes*	97.92	*Penicillium*	82.48
40,000	*Pseudomonas*	1.93	*Achromobacter*	17.43
40,000	*Meiothermus*	0.12	*Sclerotinia*	0.06
40,000	*Bradyrhizobium*	0.02	*Escherichia*	0.01
40,000	*Corynebacterium*	0.004	*Bacteroides*	0.004

### Viable microorganisms

Isolated bacterial colonies were identified by searching 16S Sanger sequences against the NCBI non-redundant database using BLASTn^22^. Table 2 summarizes the isolates. *Bacillus* was the most prevalent genus identified, measured in 25% of flight samples and all control samples. *Streptococcus* and *Streptomyces* were also identified from a 40,000 ft sample. Other bacteria found in controls were: *Lysinibacillus sinduriensis*, *Methylorubrum zatmanii*, *Lysinibacillus sphaericus*, *Lysinibacillus acetophenoni*, *Pseudomonas syringae*, *Microvirga* sp., *Methylorubrum populi*, and *Cellulomonas cellasea*.

**Table 2:** Viable microorganisms recovered from flight and control samples.

Category	Environment	Genera identified
Flight samples	10,000 ft Filter A	NA
	10,000 ft Filter B	NA
	20,000 ft Filter A	*Bacillus zhangzhouensis*^5^*^,^^+^, *Bacillus pumilus*^1^^+^
	20,000 ft Filter B	NA
	30,000 ft Filter A	*Bacillus zhangzhouensis*^2^, *Bacillus *spp.^2^^^^
	30,000 ft Filter B	NA
	40,000 ft Filter A	NA
	40,000 ft Filter B	*Streptococcus* sp.^1^^^^, *Streptomyces* spp.^2^^^^
Ground control	Pre-flight plane swab	*Bacillus subtilis*^4^^+^, *Bacillus pumilus*^3^^+^, *Bacillus megaterium*^3^^+^, *Bacillus zhangzhouensis*^1^^+^, *Bacillus safensis*^1^^+^, *Bacillus *spp.^8^, *Bacillus licheniformis*^3^^+^, *Bacillus halotolerans*^1^^+^, *Lysinibacillus sinduriesis*^1^^+^, *Methylorubrum zatmanii*^2^^^^, *Lysinibacillus *sp^1^*.* ^+^
	Post-flight plane swab	*Bacillus *spp. ^2^^+^, *Bacillus zhangzhouensis*^6^
Cabin control	Bench swab^+^	*Bacillus subtilis*^3^, *Bacillus safensis*^1^, *Bacillus maritimus*^1^, *Lysinibacillus sphaericus*^1^, *Lysinibacillus acetophenoni*^1^
	Witness R2A plate	*Pseudomonas syringae*^1^^+^, *Microvirga*^#,$^^1^^+^, *Methylorubrum populi*^$^^1^^+^, *Cellulomonas cellasea* ^1^^+^, *Streptococcus *sp ^1^^^^, *Agromyces*^#^ sp ^1^^^^, *Planomicrobium glaciei*^$^^1^^+^, *Staphylococcus epidermidis* ^2^^+^, *Bacillus licheniformis*^2^^^^, *Citrococcus *sp. ^1^^^^, *Bacillus paralicheniformis*^1^^^^
Process control^&^	PBS elution fluid control	*Bacillus zhangzhouensis* ^1^, *Bacillus *sp. ^1^

*The number in brackets indicates number of isolates matching the genus and/or species.

^#^Designates query lengths < 1,000 nt.

^$^Query sequence had < 95% identity to database match.

^+^Isolates from June 20, 2018 sampling only.

^^^Isolates from June 21, 2018 sampling only.

^&^Control sample taken in lab July 2018.

### Atmosphere dispersal

Generally, the air sampled on each flight day shared a similar transport history off the coast of N. America. Supplementary Fig. S7 provides kinematic back trajectory modeling with Hybrid Single-Particle Lagrangian Integrated Trajectory (HYSPLIT). We note that none of the trajectories above 10,000 ft approached the Earth’s surface over the time period modeled. There were also clear differences in trajectories between samples at the same altitude separated by approximately 24 h.

**Figure 5:**
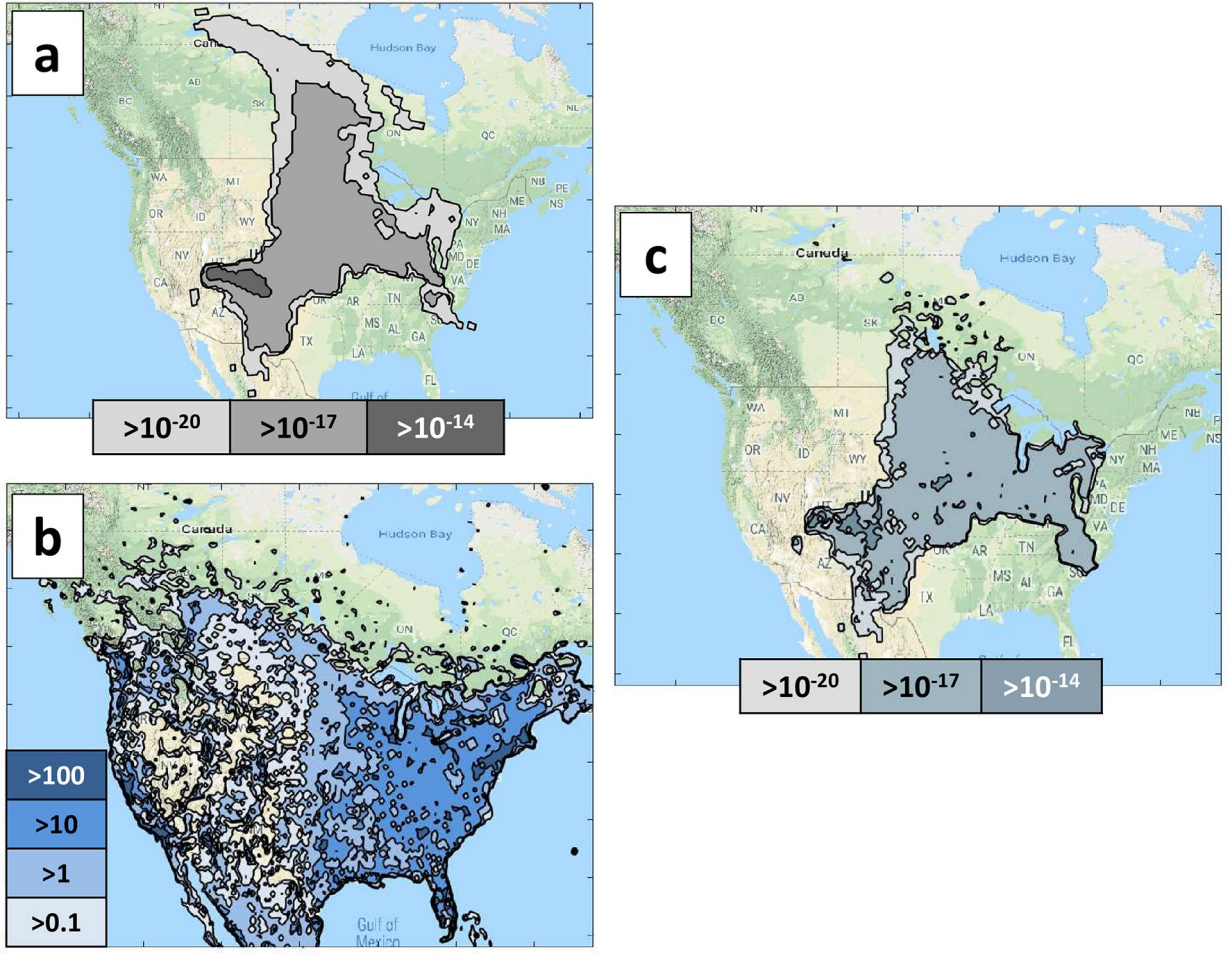
Aerosol dispersal predictions. (**a**) 7-day model forecast (June 20, 2018 to June 27, 2018) with grey contours showing fraction of sampled material that could have been inhaled by an average outdoor individual (individual exposure potential); (**b**) The blue contours show the population density (people per sq km) in the modeling domain; (**c**) The blue-grey contours show the population level exposure potential derived by combining individual exposure potential (shown in *a*) with population estimates (shown in *b*). This image shows geographic distribution of potentially inhaled material (inhaled fraction of sample material per sq km). Panels were generated using Mathworks Matlab software, versions R2018 to R2019 (https://www.mathworks.com/products/matlab.html), from the modelling described in the text. Underlying map data was provided by Google and INEGI and accessed through the Google Maps Platform API.

Separate, forward-modeling of air masses showed that a portion of flight-sampled bioaerosols, if undisturbed, could have exposed outdoor downwind populations over the next week (Fig. 5). In this figure, the grey contours indicate the fraction of sampled material that would have been inhaled by an average outdoor person (individual exposure) had the material not been sampled (termed potential exposures). Darker colors indicate a larger potential exposure. Figure 5a shows the sum of the potential exposures from all four samples (10,000 ft, 20,000 ft, 30,000 ft and 40,000 ft). Note that the exposure may not occur uniformly over the week period, but rather the exposure regions can change as the air mass travels downwind and is diluted, see Fig. 5b,c. The predicted potential impact areas were highly dependent on altitude. For instance, most of the impacts in the first week were associated with air sampled at 10,000 ft while air sampled at 20,000 ft was associated with a small potential exposure region in NV (USA). No potential exposures were associated with the highest altitude samples (30,000 and 40,000 ft) during the first week. Overall, only a small fraction (3 × 10^−8^) of sampled particles would be expected to reach population centers across North America and be inhaled during the first week. The actual amount of material inhaled would be smaller than predicted here since (a) most people are indoors and (b) indoor individuals have lower exposure, relative to outdoor individuals, to outdoor-origin airborne particulates^32,33^.

## Discussion

With an aerobiology study flown into the upper troposphere, we detected changing microbial abundance with height above the Earth’s surface – our dataset was reliant upon a shotgun metagenomics approach analyzing samples acquired between 10,000 to 40,000 ft, on two consecutive summer days over the US Sierra Nevada mountain ranges. Controls were also sequenced as a baseline reference. This strategy was essential because contaminants can confuse signals from low biomass environments. We observed significant differences in microbial taxa from flight samples compared to control samples. The most abundant taxa identified in the flight samples were: *Achromobacter, Alcaligenes, Delftia, Lachnoanaerobaculum, Mycobacterium, Penicillium, Prevotella, Pseudoperonospora, Stenotrophomonas, Streptococcus, Tetrahymena,* and *Veillonella. Alcaligenes* is a genus of gram-negative, aerobic bacteria found mostly in the intestinal tracts of vertebrates, decaying materials, dairy products, water, and soil. It can cause opportunistic infections, including nosocomial sepsis. *Alcaligenes faecalis* is usually found in soil and water environments, and its abundance in atmospheric samples hints at a possible relationship with agriculture (e.g., wastewater, compost, feedlots^34^) considering the physical proximity of air sampled to the CA Central Valley with ~ 28,000 km^2^ of irrigated farmlands. Up to 55% of dust deposition at alpine sites in the Sierra Nevada comes from the Central Valley^35^; thus, the air sampled in this study would be expected to contain agriculturally-produced bioaerosols. The proportional abundance of *Stenotrophomonas maltophilia* identify it as another candidate to examine for possible emanation from the Central Valley, considering the species is regularly found in plant rhizospheres, animals, croplands, and water sources^36^. Other noteworthy bacterial standouts in our atmospheric samples—part of the normal flora of the mouth and gastrointestinal tract in humans—were *Delftia acidovorans*^37^, *Lachnoanaerobaculum saburreum*^38^, *Prevotella*, *Veillonella* and *Streptococcus*. Wastewater treatment facilities could be a contributing source of these bioaerosols^39^. *Mycobacterium* was among the most abundant genera at 10,000 ft on the first sampling day; aerosolized nontuberculous mycobacteria can cause pulmonary disease^40^, but we did not produce viability information with DNA-based detections. *Achromobacter xylosoxidans* may be the most clinically relevant species detected in our study (endogenous microbiota of the ear and gastrointestinal tract^41^); however, considering it was also measured in control samples, this detection may reflect contamination*.* Two fungal species detected among the most abundant taxa (*Pseudoperonospora* and *Penicillium*) are of particular note. *Pseudoperonospora cubensis* is a fungal pathogen with a wide geographical distribution that can infect produce including cantaloupe, cucumber, pumpkin, squash and watermelon^42^. Similarly, *Penicillium aurantiogriseum* found in our dataset, can cause spoilage of various food products^43^.

Most previous high altitude aerobiology studies have lacked rigorous control measurements, making it difficult to determine whether taxa reported came from the atmosphere (native biomass) or the sampling systems (false positives). Accordingly, in this study we followed the protocol described^44^ where a comprehensive set of controls were reported to provide a transparent picture of baseline contamination associated with the study. The approach, summarized in brief, included descriptions of: (1) sterilization methods; (2) ground transportation controls; (3) hardware controls; (4) laboratory/assay controls; and (5) any contamination results measured. With the improved sensitivity of most molecular methods, including the DNA-based detection assays reported herein, some level of baseline contamination will always be expected for environmental and laboratory controls. Consequently, our study relied upon data analysis techniques that showed the “signal-to-noise”, including ordination plots comparing how environmental (atmospheric) samples clustered significantly apart from the sampling system or laboratory control samples (Supplementary Table S2 and Fig. S6). Of course, the analysis approach also identified common taxa across environmental and control groups; for example, *Achromobacter xylosoxidans* and *Bradyrhizobium* sp. DFCI-1 where the two species were detected in both flight samples and control samples. In this case, the overlap indicates likely contamination so *Achromobacter xylosoxidans* and *Bradyrhizobium* sp. DFCI-1 were probably not sampled from the atmosphere.

Viable bacteria measured in our study (e.g., *Bacillus zhangzhouensis*, *Bacillus pumilus*) included some endospore formers resistant to extremes and frequently reported in other high altitude surveys^2,3^. Using high altitude balloons for collecting bioaerosols up to 125,000 ft (38 km), Actinobacteria, Firmicutes, and Proteobacteria isolates were recovered^45^. Ground-based simulations using the stratospheric isolates estimated that some bacteria could survive long-range transport in the atmosphere up to 140 days if shaded from direct sunlight^45^. It is therefore plausible that many other viable (but non-cultured) microorganisms can be identified in this metagenomics dataset.

The long-range dispersal of viable bioaerosols may be critical to understanding agricultural and public health implications because the troposphere and lower stratosphere can enable microbial dispersal over geographic barriers^3^. Most knowledge in this emerging area of aerobiology comes from regional studies. Soybean rust, for instance, has been correlated with incoming dust storms^46^. Valley Fever outbreaks in the Southwest of US is thought to be caused by dust storms carrying a fungal pathogen, *Coccidioides immitis*^47^. Desert storms in sub-Saharan Africa have also been linked to outbreaks of a deadly bacterium, *Neisseria meningitides,* affecting 26 countries and more than 300 million people. This same region, also called *“*the Meningitis belt”^47^, is also prone to epidemics such as malaria. Interestingly, we measured a strong DNA signature of *Tetrahymena* (first flight day at 10,000 ft), a ciliated protozoan typically found in aquatic ecosystems. No previous aerobiology survey has detected airborne protozoa at such heights above the Earth’s boundary layer. Another unexpected DNA signal was *Pinus* (second flight day at 10,000 ft), suggesting the sampled air mass had recently passed over forested terrain in the US Sierra Nevada mountains – taken together with the back trajectory model at 10,000 ft, the result might demonstrate how orographic uplift connects regional surface emissions with the upper atmosphere.

Across two consecutive sampling days, the atmospheric microbiome was dynamically changing at each altitude. *Lachnoanaerobaculum*, *Alcaligenes, Mycobacterium*, *Tetrahymena,* and *Veillonella* were abundant on the first day, while *Penicillium, Prevotella, Pseudoperonospora,* and *Porphyromononas* were only enriched on the second day. Such temporal variability is common in other aerobiology reports and is further supported by the different back-trajectories of the two days (Supplementary Fig. S7). A recent study examined how local topography and wind conditions can influence regional bioaerosol dynamics. *Bacillus* and *Sphingomonas* (for bacteria) and *Pseudotaeniolina globaosa* and *Cladophialophora proteae* (for fungi) were the most abundant taxa detected, but the authors observed relative abundance varying at disparate sampling locations^12^. Similarly, another study showed that diurnal cycles in the boundary layer resulted in fungal and bacterial aerosols shifting with temperature, humidity, and CO_2_ conditions^48^. Both dry and wet deposition significantly influence bioaerosol patterns and it is worth emphasizing our flights occurred in non-cloudy, precipitation-free areas^49^. With a multiyear campaign lasting seven years at an alpine field site in Spain, dynamic, seasonal shifts in 16S rRNA measurements (yielding mostly Alphaproteobacteria and Betaproteobacteria) were found^4^. One bacterial genus showing up in high relative abundance in both our study and that of the study in Spain^4^ was *Stenotrophomonas*. Actinobacteria, Firmicutes, Bacteroidetes, and Proteobacteria are other common phyla between our results and other vertically-sampled air masses over desert regions in Asia^50^.

Most previous vertical aerobiology studies relied upon 16S rRNA amplicon methods, which targets and PCR-amplifies short regions of the 16S gene, for characterizing bacteria sampled^4,12,45,50,51^, whereas our shotgun metagenomics approach has the capacity to detect a broader taxonomic range with higher resolution, to the species or strain/plasmid level. Metagenomic sequencing provides relative quantitation based on the number of reads for each taxon identified; but it does not provide absolute DNA concentrations. Producing more accurate human, animal, and plant impact forecasting models will benefit from quantitative information about abundance (e.g., DNA concentration or cell counts) and, to a lesser extent, the size distribution of bioaerosols. For now, we rely upon other datasets for quantitative information that we were unable to acquire in this study. Depending on the altitude sampled, in previous reports of airborne bacteria, the bacteria concentration ranged from 10^3^ to 10^7^ cells·m^−3^ over 3,300 to 26,000 ft (1–8 km)^51^. Separately, it was found that total airborne bacterial concentrations at 9,800 ft (3 km) were similar to values at 95,000 ft (29 km), approximately 5 × 10^5^ cells·m^−3^^45^. Another study examined the size distribution of bioaerosols associated with near-surface pollution, and similar research will be needed to more robustly characterize microorganisms reaching the upper atmosphere^52^. One challenge with using the ABC system onboard the C-20A is that the geometry of the aerosol inlet likely prevents most large aerosols (> 4 µm) from reaching the collector^2^. Therefore, more efficient, size-inclusive sampling methods will need to be developed in future years, in addition to including instruments that can be useful for characterizing atmospheric species in real-time as well as provide useful information for use in fate, transport, and exposure modeling^8^.

An uptick of international aerobiology studies is finally allowing global atmospheric microbiome patterns to be examined. However, the surge of recent literature also underscores the importance of developing consistent sampling and analysis methodologies^11^. One of the primary goals of our work was to demonstrate that vertical bioaerosol measurements could be made across the troposphere using the most sensitive molecular methods available. Our metagenomics dataset and modeling analysis provides a more inclusive framework for planning field campaigns that will someday make concurrent measurements at emission sources and downwind locations. In the Central Valley, for instance, bioaerosols picked up by easterly winds, heading towards the US Sierra Nevada mountain range, might be sampled near the source (adjacent to agricultural fields), in the air (using aircraft), and on the ground (at alpine field observatories). Such multipronged efforts may result in refined forecast models for winds carrying potentially disruptive pathogens.

### Accession codes

The raw FASTQ files are available in the NASA GeneLab repository under the accession number GLDS-256.

## Supplementary information

Supplementary information
