# Supplementary material for: Sierra Nevada sweep: metagenomic measurements of bioaerosols vertically distributed across the troposphere
Source: Sci Rep. 2020 Jul 24;10:12399. doi: 10.1038/s41598-020-69188-4 (PMC7382458; doi:10.1038/s41598-020-69188-4)

Sierra Nevada sweep: Metagenomic measurements of bioaerosols vertically distributed across the troposphere

Crystal Jaing[1,*], James Thissen[1], Michael Morrison[1], Michael B. Dillon[1], Samantha M. Waters[2,5], Garrett T. Graham[3], Nicholas A. Be[1], Patrick Nicoll[4], Sonali Verma[5], Tristan Caro[6] and David J. Smith[7]

[1] Lawrence Livermore National Laboratory, Livermore, CA, USA

[2] Universities Space Research Association, USA

[3] Georgetown University Medical Center, Washington, DC, USA

[4] University of Victoria, BC, Canada

[5] Blue Marble Space Institute of Science, NASA Ames Research Center, Space Biosciences Division, Moffett Field, CA, USA

[6] Interdisciplinary Quantitative Biology, BioFrontiers Institute, University of Colorado, Boulder, CO

[7] NASA Ames Research Center, Space Biosciences Division, Moffett Field, CA, USA

*Corresponding author: jaing2@llnl.gov

Supplementary figure legends

Supplementary Fig. S1. Flight telemetry data over US Sierra Nevada range. (a) June 20, 2018; **(b)** June 21, 2018. Telemetry files (.*kmz* format) from the two flights were visualized using Google Earth Pro software v7.3.2.5776 (64-bit) (https://www.google.com/earth/versions/#earth-pro). Google Earth map data was accessed and annotated using Google Earth Pro software on February 3rd, 2019. Coordinates 34°57'00.0"N 117°53'14.2"W, the coordinates of Armstrong Flight Research Center, were used to center the map.

Supplementary Fig. S2. The two-stage cascade impactor (Product TE-10-860; Tisch Environmental, Cleves, OH). (a) The cascade impactor; (b) The first stage of the impactor with small round drilled orifices that are 1.18 mm; (c) the second stage of the impactor with small round drilled orifices that are 0.25mm.

Supplementary Fig. S3. Alpha (α)-diversity for flight and ground samples at species level resolution. α-diversity quantified as $^{1}N_{eff}$ with samples shown as circles. The area of each circle is proportional number of species-resolved fragments in a sample. Violin plots show density of α-diversity, box plots show the first, second and third quartiles, and 1.5 times the interquartile range of each time point's α-diversity sample distribution. Dark red diamonds represent the means. The figure was generated in R[1] using package ggplot2 (v.3.2.1)[2].

Supplementary Fig. S4. Relative abundance of genera across altitudes on both sampling days. Samples from Filter B separated by day and collection altitude to examine shifts in relative

---

[1] R: A Language and Environment for Statistical Computing v. 3.6.0 (R Foundation for Statistical Computing, Vienna, Austria, 2019).
[2] H. Wickham. ggplot2: Elegant Graphics for Data Analysis. Springer-Verlag New York, 2016.

abundance of genera. Bacterial genera that were not among the top 12 represented by light gray colored bars. The figure was generated in R[1] using package ggplot2 (v.3.2.1).

Supplementary Fig. S5. Relative abundance of species across altitudes on both sampling days. Samples from Filter A separated by day and collection altitude to examine shifts in relative abundance of species. *Alcaligenes* faecalis, *Delftia sp*. Cs1-4*, Penicillium aurantiogriseum, Pseudoperonospora cubensis* and *Stenotrophomonas maltophilia* were among the top 12 most abundant taxa not also detected in control samples. Bacterial species that were not among the top 12 represented by light gray colored bars. The figure was generated in R[1] using package ggplot2 (v.3.2.1).

Supplementary Fig. S6. Relative abundance of species in experimental control samples. Influences of potential contaminants associated with aircraft sampling method. Bacterial species that were not among the top 12 represented by light gray colored bars. The figure was generated in R[1] using package ggplot2 (v.3.2.1).

Supplementary Fig. S7. Kinematic back trajectories showing transport history of air masses sampled. On both days (June 20-21, 2018) and at all altitudes sampled, HYSPLIT[3] models indicated air traveling in an easterly direction. The authors gratefully acknowledge the NOAA Air Resources Laboratory (ARL) for the provision of the HYSPLIT transport and dispersion model and/or READY website (https://www.ready.noaa.gov) used in this publication.

---

[3] Stein, A.F., Draxler, R.R, Rolph, G.D., Stunder, B.J.B., Cohen, M.D., and Ngan, F., (2015). NOAA's HYSPLIT atmospheric transport and dispersion modeling system, Bull. Amer. Meteor. Soc., **96**, 2059-2077, http://dx.doi.org/10.1175/BAMS-D-14-00110.1

Supplementary Table S1. Start and finish time (UTC) of sample collection at the four different altitudes

	June 20			June 21		
**Altitude (ft)**	Start time	End time	Duration (min)	Start time	End time	Duration (min)
**40,000**	21:18	21:48	40	19:05	19:35	30
**30,000**	21:54	22:24	30	19:42	20:12	30
**20,000**	22:32	23:02	30	20:20	20:50	30
**10,000**	23:08	23:38	30	21:02	21:32	30

Supplementary Table S2. Samples types and number of reads obtained from each sample.

Sample internal label	Sample Labels	Illumina reads	Total LMAT reads	Microbe LMAT reads	Genus level reads	Filter	Sample details
**ABC2_Amp_7**	Sample 1	16786136	571271	118917	4033	alpha	6/20/2018 10K filter
**ABC2_Amp_8**	Sample 2	15641786	7293844	175042	33066	beta	6/20/2018 10K filter
**ABC2_Amp_9**	Sample 3	14053300	4671920	1145585	468155	alpha	6/20/2018 20K filter
**ABC2_Amp_10**	Sample 4	13983138	6949492	217662	12917	beta	6/20/2018 20K filter
**ABC2_Amp_11**	Sample 5	14151057	7048196	144915	984	alpha	6/20/2018 30K filter
**ABC2_Amp_12**	Sample 6	10691675	5313980	209218	89119	beta	6/20/2018 30K filter
**ABC2_Amp_13**	Sample 7	20567555	7866853	2958182	1445418	alpha	6/20/2018 40K filter
**ABC2_Amp_14**	Sample 8	11673654	3820724	76775	5226	beta	6/20/2018 40K filter
**ABC2_Amp_20**	Sample 9	8842209	4403916	66611	2198	alpha	6/21/2018 10K filter
**ABC2_Amp_21**	Sample 10	16909991	7806565	86447	47854	beta	6/21/2018 10K filter
**ABC2_Amp_22**	Sample 11	10560451	4795645	127547	1655	alpha	6/21/2018 20K filter
**ABC2_Amp_23**	Sample 12	10492344	4678165	4268366	4114235	beta	6/21/2018 20K filter
**ABC2_Amp_24**	Sample 13	12432228	6170148	1754529	437378	alpha	6/21/2018 30K filter
**ABC2_Amp_25**	Sample 14	10234886	5071097	2472976	2277788	beta	6/21/2018 30K filter
**ABC2_Amp_26**	Sample 15	11092099	5308798	1136116	838540	alpha	6/21/2018 40K filter
**ABC2_Amp_27**	Sample 16	11013130	5327841	110915	29643	beta	6/21/2018 40K filter
**ABC2_Amp_16**	Ground Control 1	10947078	2030941	78629	2607	pre	6/20/2018 Pre-flight probe swab
**ABC2_Amp_17**	Ground Control 2*	10199659	540058	418549	305132	pre	6/20/2018 Pre-flight ground swab
**ABC2_Amp_29**	Ground Control 3	12412853	6036342	1442837	992322	pre	6/21/2018 Pre-flight probe swab
**ABC2_Amp_30**	Ground Control 4	11091806	5473440	643993	53574	pre	6/21/2018 Pre-flight plate swab
**ABC2_Amp_31**	Ground Control 5	10243693	4507476	100530	30256	pre	6/21/2018 Pre-flight ground swab
**ABC2_Amp_19**	Ground Control 6	12105196	5523686	3630255	3350744	post	6/20/2018 Post-flight ground swab
**ABC2_Amp_32**	Ground Control 7	12694676	4251879	89322	6670	post	6/21/2018 Post-flight ground swab
**ABC2_Amp_15**	Cabin air Control 1	294	43	6	1	NA	6/20/2018 Blank filter witness
**ABC2_Amp_18**	Cabin air Control 2	15061699	6367162	786202	40247	NA	6/20/2018 In-flight bench swab
**ABC2_Amp_28**	Cabin air Control 3	12150405	6046165	591102	1687	NA	6/21/2018 Blank filter witness
**ABC2_Amp_3**	Process Control 1	21767517	10255327	2689397	116636	NA	Whole blank gelatinous membrane filter that has gone through the whole process to extraction
**ABC2_Amp_33**	Process Control 2	1695050	600850	239053	12	NA	7/27/2018 Powerviral Extraction NTC
**ABC2_Amp_34**	Process Control 3	1538580	766615	200810	37447	NA	7/30/2018 Powerviral Extraction NTC

*Ground control 2 was an outlier from the rest of the ground control samples, therefore not used in generating Figures 2 and 3.

Supplementary Fig. S1

a

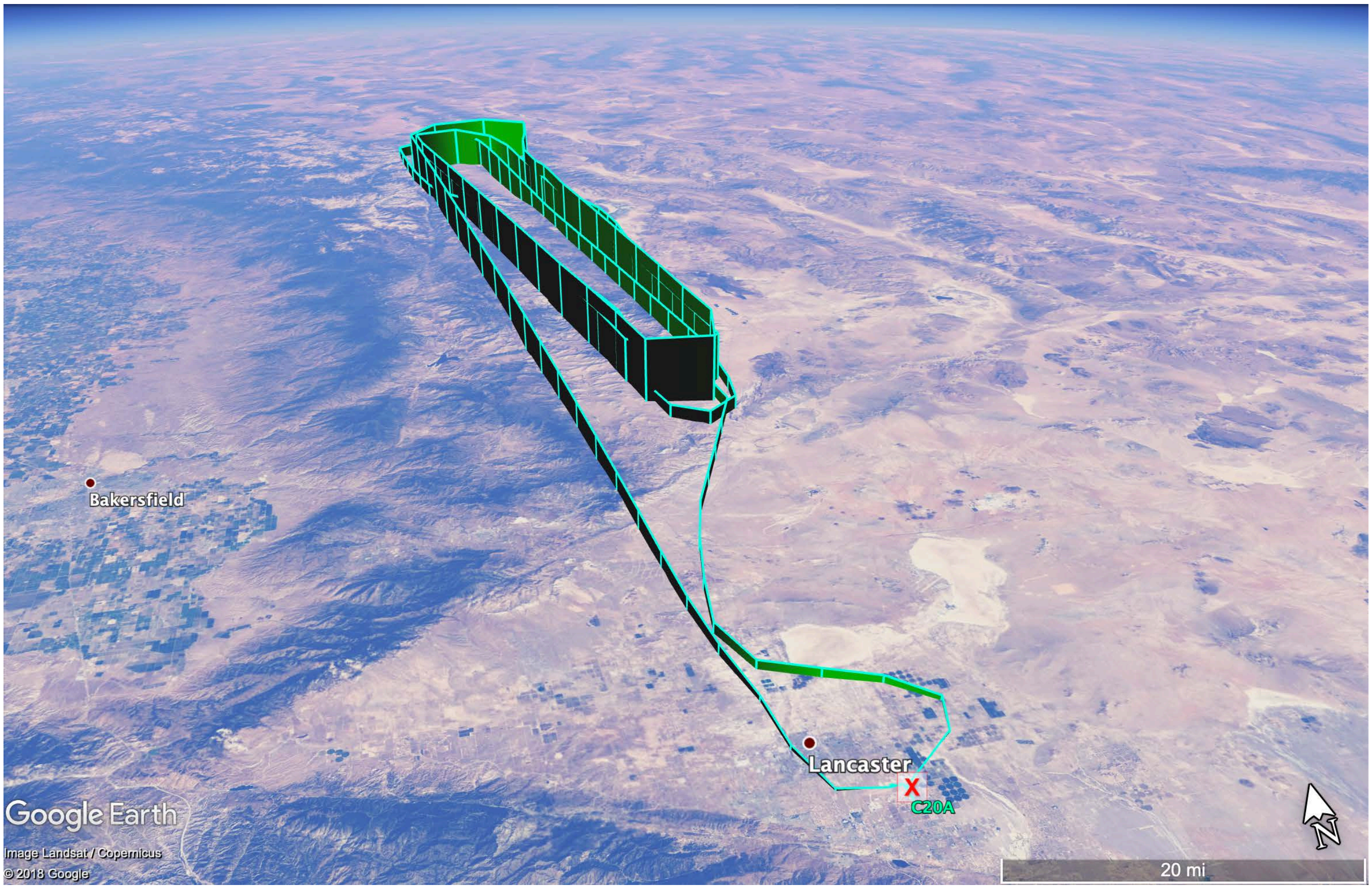

b

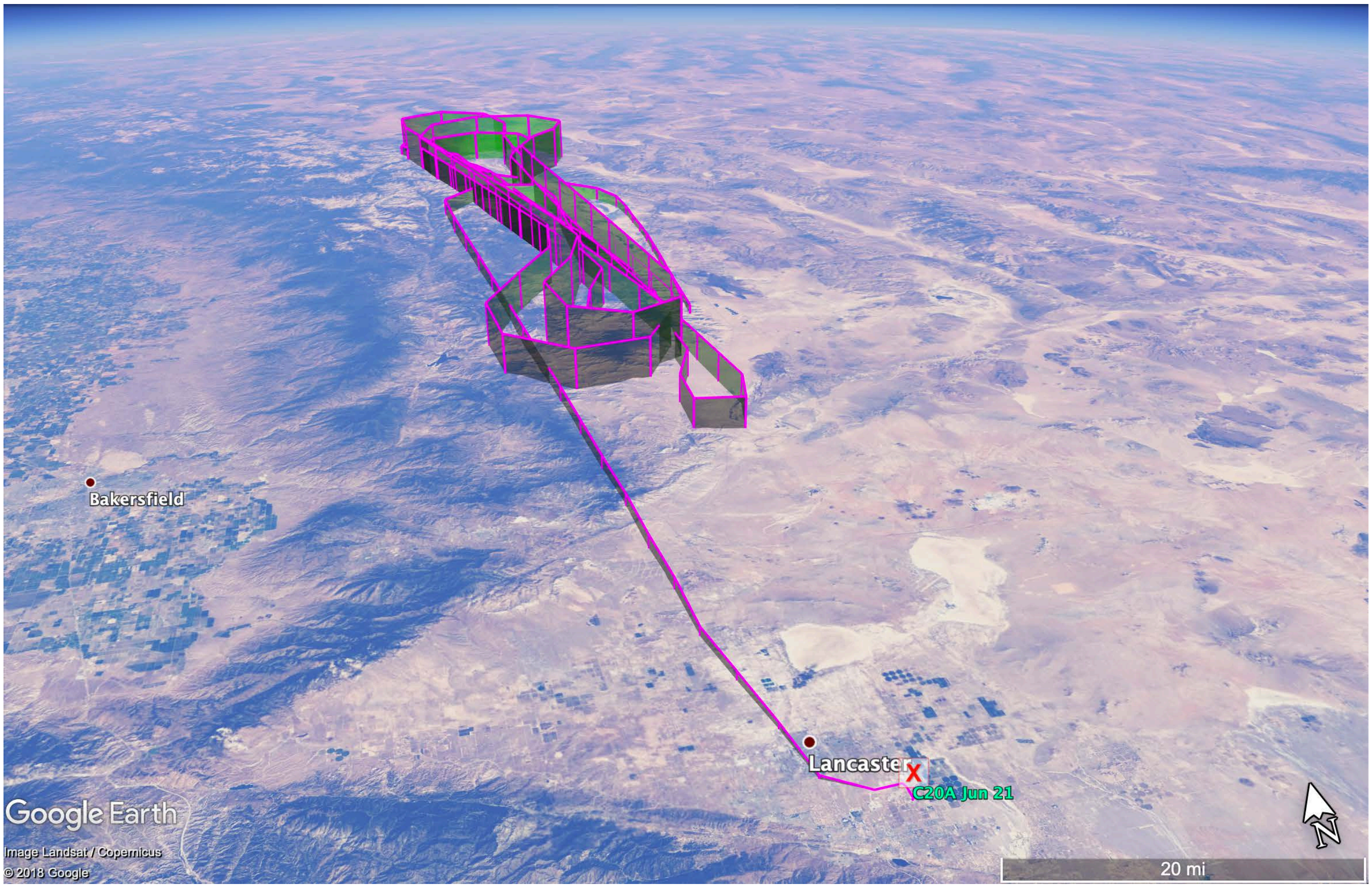

Supplementary Fig. S2

a b c

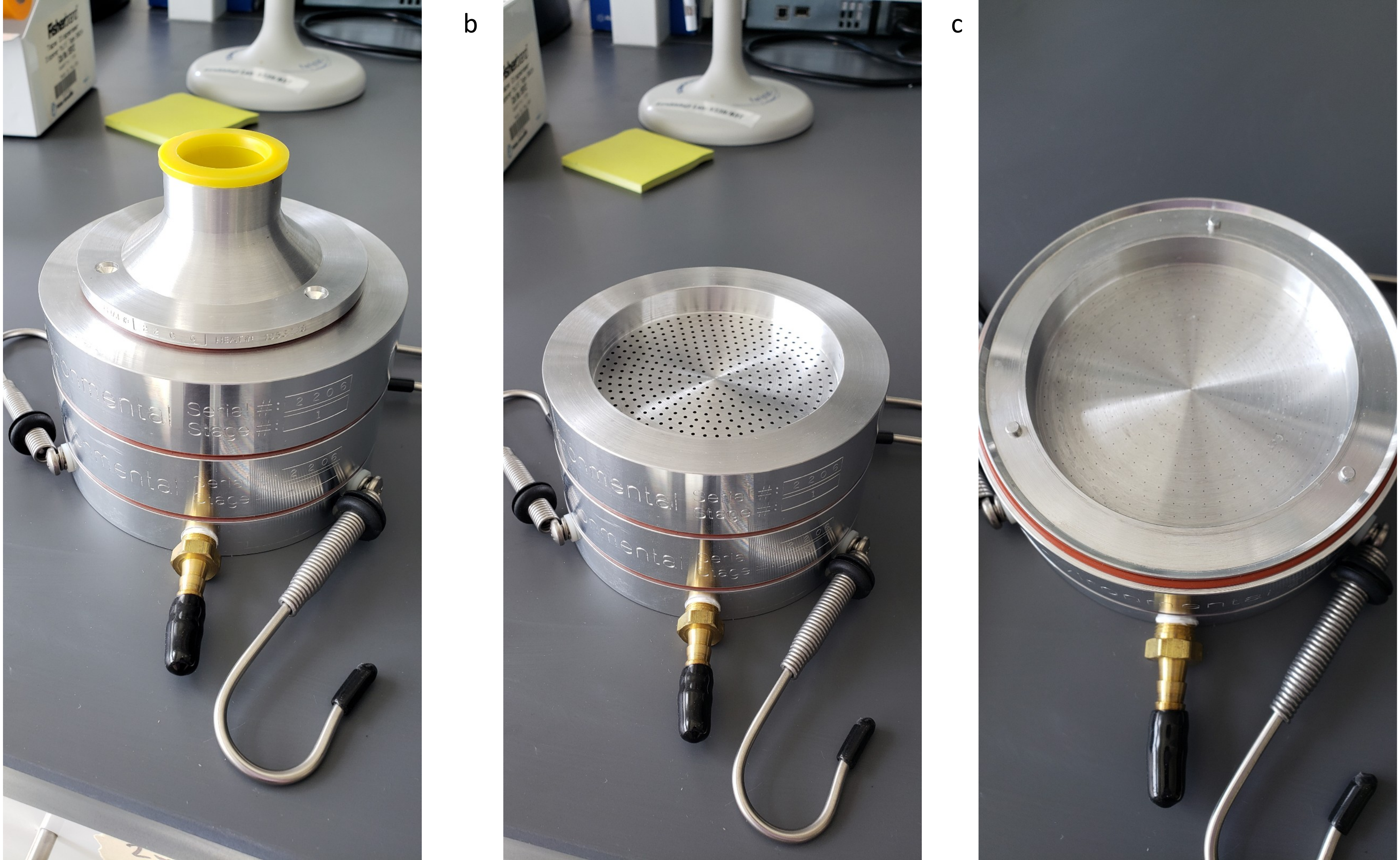

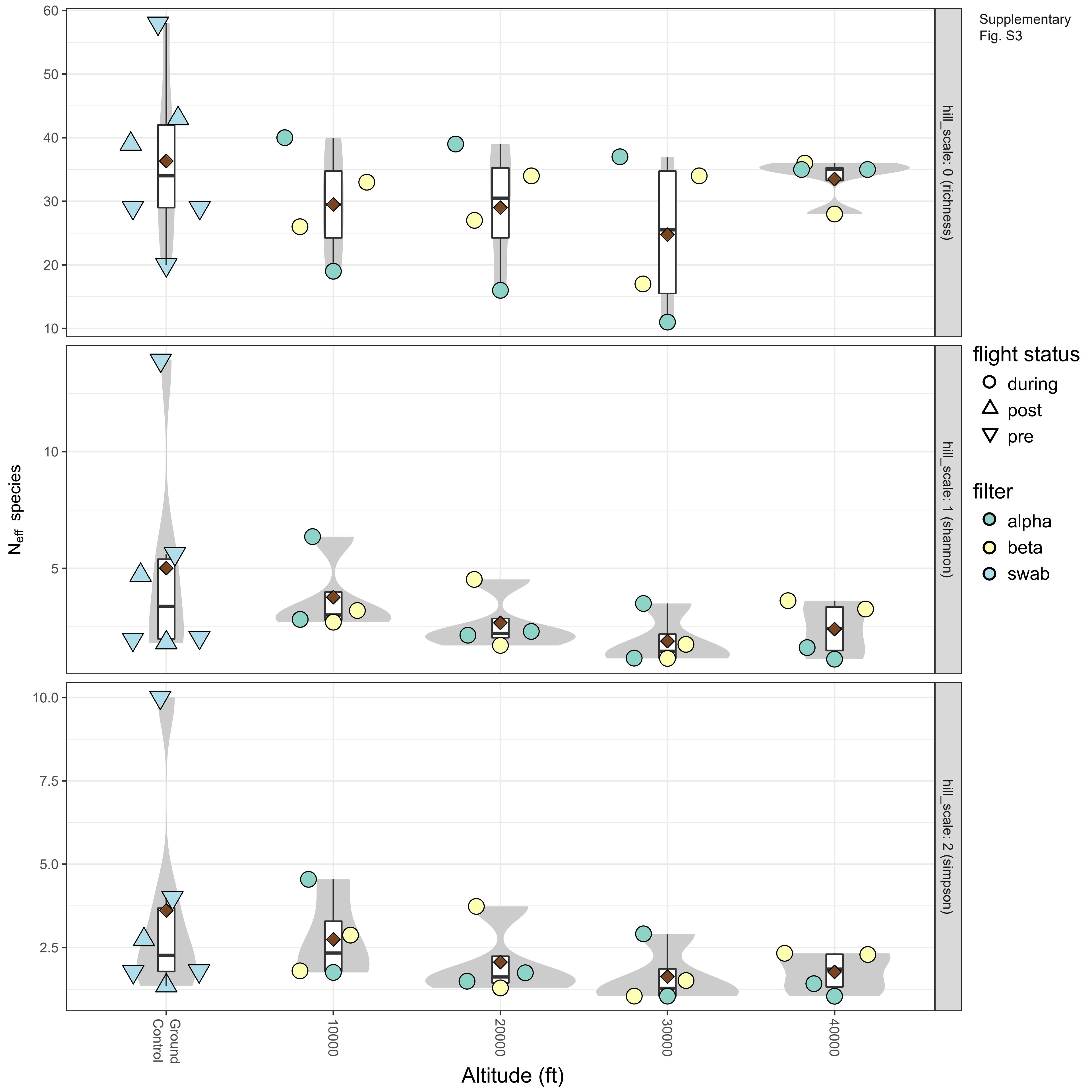
Supplementary
Fig. S3
hill_scale: 0 (richness)
hill_scale: 1 (shannon)
hill_scale: 2 (simpson)
N_{eff} species
Altitude (ft)
Ground Control
10000
20000
30000
40000
flight status
during
post
pre
filter
alpha
beta
swab

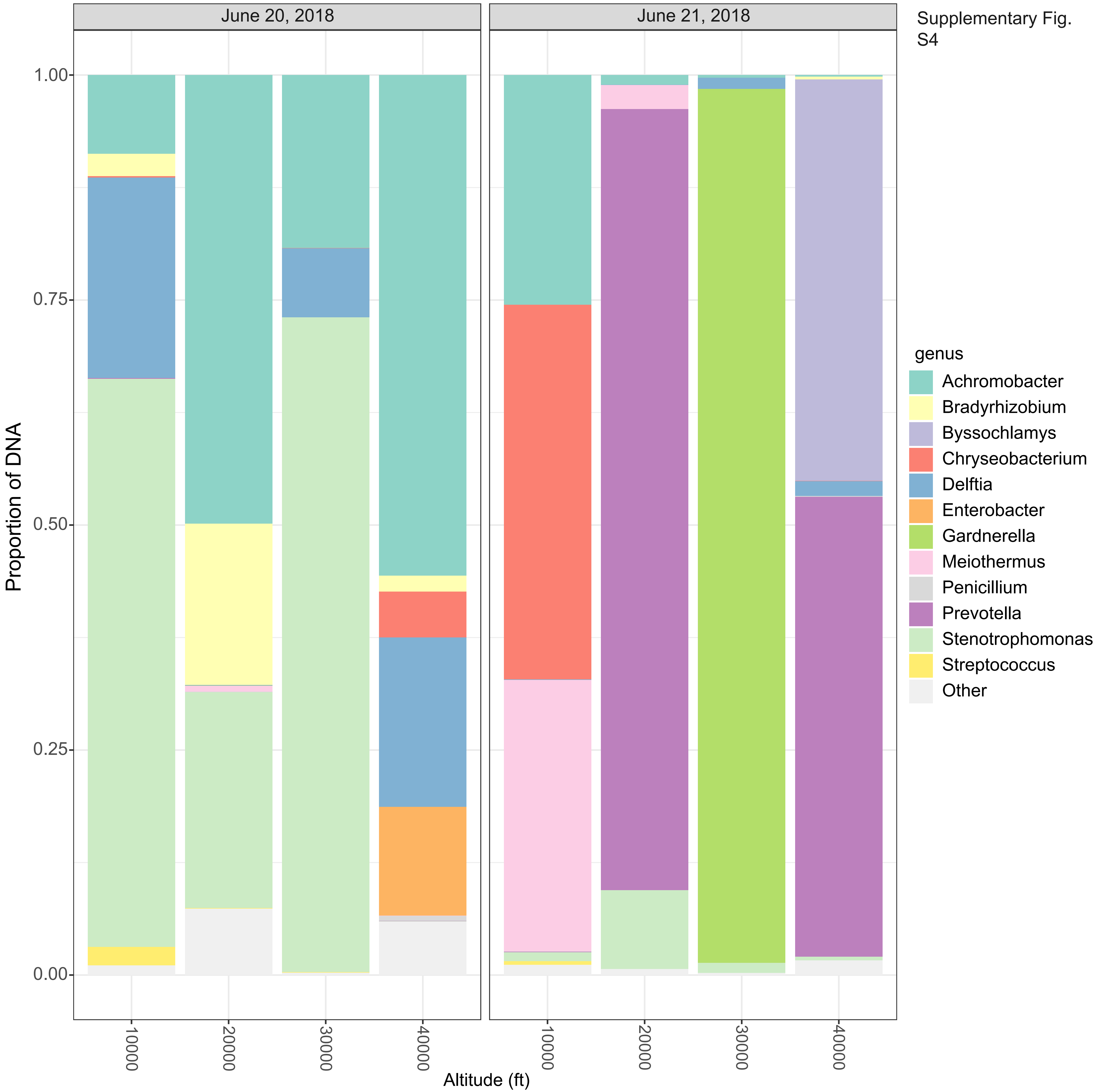

Supplementary Fig. S4

Supplementary Fig. S5

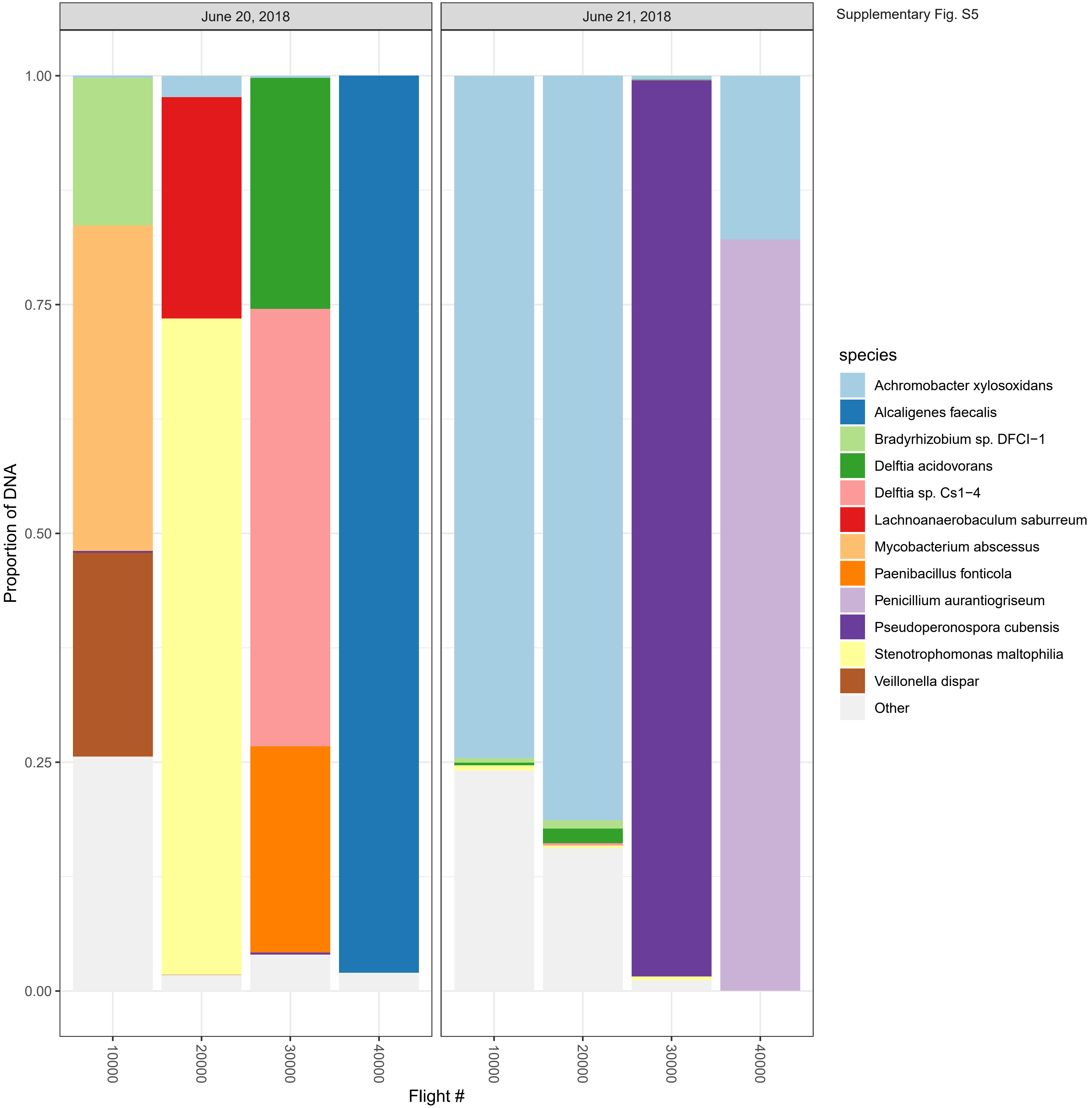

Supplementary Fig. S6

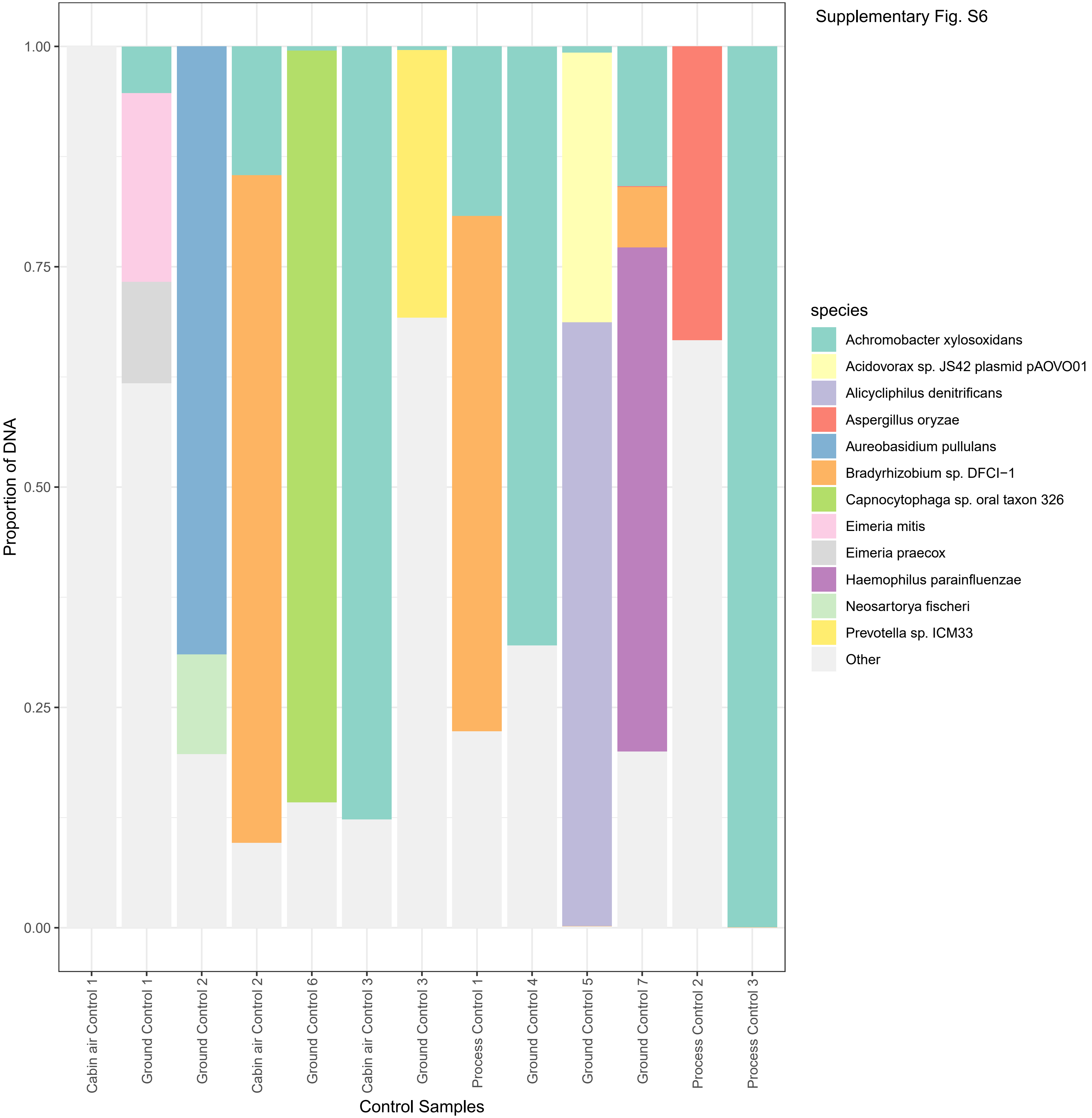

Supplementary Fig. S7

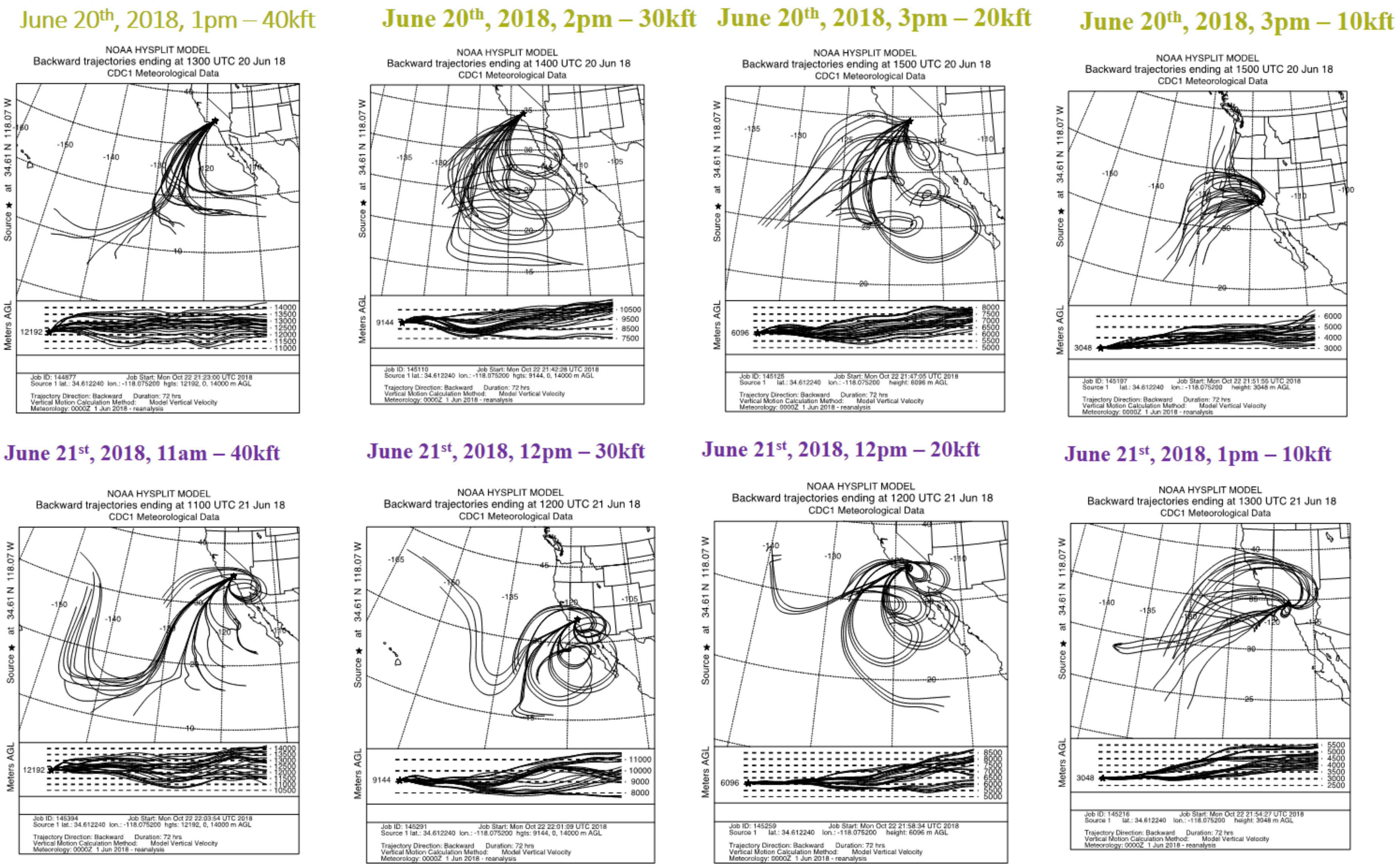

Supplement: Supplementary file 1 — Supplementary information [file 41598_2020_69188_MOESM1_ESM.pdf]
